# Relapsing Fever–Like Spirochetes Infecting European Vector Tick of Lyme Disease Agent

**DOI:** 10.3201/eid0906.020459

**Published:** 2003-06

**Authors:** Dania Richter, Daniela B. Schlee, Franz-Rainer Matuschka

**Affiliations:** * Institut für Pathologie, Charité, Humboldt-Universität zu Berlin, Berlin, Germany

**Keywords:** spirochetes, relapsing fever, Lyme disease, hard ticks, genospecies, *Ixodes ricinus*, research

## Abstract

To determine whether relapsing fever–like spirochetes associated with hard ticks may infect *Ixodes ricinus* ticks in central Europe, we screened questing ticks for 16S rDNA similar to that of Asian and American relapsing fever–like spirochetes. We compared the prevalence of these spirochetes to that of Lyme disease spirochetes transmitted by the same vector. Relapsing fever-like spirochetes infect 3.5% of questing vector ticks in our three central European sites near the Rhein Valley. These spirochetes differ genetically from their American and Asian analogs while being relatively homogeneous in the region we sampled. The Lyme disease genospecies most commonly detected in central Europe are distributed broadly, whereas those that are less frequently found appear to be place-specific. The absence of co-infected ticks suggests that relapsing fever–like and Lyme disease spirochetes may not share hosts. Exposure risk for relapsing fever–like spirochetes is similar to that of certain Lyme disease genospecies. Although many persons may be bitten by ticks infected by relapsing fever–like spirochetes, health implications remain unknown.

Diverse pathogens infect ticks of the *Ixodes ricinus* complex in North America, Asia, and Europe. Viral agents include those of tick-borne encephalitis, Russian spring-summer encephalitis, louping ill, Powassan encephalitis (*1*), and its variant known as deer tick virus (*2*). The piroplasms that infect these ticks include *Babesia divergens*, *B. microti*, *B. odocoilei*, and *B.* *gibsoni* (*3*). Other infectious agents include *Ehrlichia (Anaplasma) phagocytophila*, *Rickettsia helvetica*, possibly *Bartonella quintana*, and a nonpathogenic endosymbiontic rickettsia (*4*–*6*). Entomophagic fungal, nematode, and wasp pathogens are also found in these ticks (*7*). The diverse array of Lyme disease spirochetes infecting ticks in the *I. ricinus* complex has been subdivided into at least six known genospecies, *Borrelia burgdorferi* sensu stricto, *B. afzelii*, *B. garinii*, *B. valaisiana*, *B. bissettii*, and *B. lusitaniae* (*8*,*9*). More recently, a relapsing fever–like spirochete, *B. miyamotoi* (*10*), has been added to this array of parasitic microbes.

*B. miyamotoi* was originally described from *I. persulcatus* ticks sampled in Japan. Its flagellin and 16S rRNA sequences resemble those of the relapsing fever spirochetes more closely than those of any of the known Lyme disease spirochetes (*10*,*11*). A closely related agent, designated as *Borrelia* nov. sp., has recently been discovered in North America (*12*). The primers used to detect infection by Lyme disease spirochetes generally fail to amplify DNA from these organisms. Although *Borrelia* nov. sp. infects approximately 2% of the American *I. scapularis* ticks, Lyme disease spirochetes are found in approximately 12%. The prevalence of *B. miyamotoi* infection in Japan, however, has not been determined. Whether these potential threats to human health are present in Europe remains unknown.

Relapsing fever–like spirochetes associated with hard ticks may infect European *I. ricinus* ticks. To determine whether such organisms are present in Europe, we screened questing ticks for DNA similar to that of the American and Asian relapsing fever–like spirochetes. In particular, we amplified a fragment of the 16S rRNA gene found in the various genospecies of Lyme disease spirochetes as well as in the Asian and American relapsing fever–like spirochetes and estimated how prevalent such infections are in a region in central Europe.

## Materials and Methods

We sampled questing *I. ricinus* ticks from three sites located near the Rhein Valley in Germany and France. The German site was near the town of Maikammer and the French site was near the town of Lembach, approximately 40 km southwest of Maikammer. A second French site, Petite Camargue Alsacienne, was situated 160 km farther south, near the German and Swiss borders (*13*). Ticks were collected in April 2001 by passing a flannel flag over vegetation. Ticks were confined in screened vials and stored at 10°C ± 1°C and all nymphs and adults were identified to species.

To detect and identify the various Lyme disease spirochetes and relapsing fever–like spirochetes in these ticks, the opisthosoma of each was opened in a drop of physiologic saline, and the midgut was obtained and transferred to a tube containing 180 μL lysis buffer (ATL Tissue Lysis Buffer, Qiagen QmbH, Hilden, Germany) and 20 μL proteinase K (600 mAU/mg). Midguts were lysed at 56°C overnight. DNA was extracted by using the QIAamp DNA Mini Kit (Qiagen QmbH) according to the manufacturer’s instructions. DNA of nymph or adult ticks was eluted with 50 μL or 75 μL elution buffer, respectively, and stored at –20°C until polymerase chain reaction (PCR) was performed.

*Borrelia* genospecies were characterized by amplifying and sequencing a 600-nucleotide fragment of the gene encoding the 16S rRNA. To increase the sensitivity for spirochetal DNA detection in ticks, we used nested PCR. Aliquots of DNA suspensions (2 μL) were diluted to 50 μL by using 200 μM of each deoxynucleoside triphosphate, 1.5 mM MgCl_2_, 0.5 U Taq polymerase (Qiagen QmbH) as well as 15 pmol of the outer primer pair and PCR-buffer supplied with the Taq polymerase. We used the following primer sequences of the 16S rRNA gene as outer primers (*14*): 16S1A 5′-CTA ACG CTG GCA GTG CGT CTT AAG C-3′ and 16S1B 5′-AGC GTC AGT CTT GAC CCA GAA GTT C-3′ (positions 36–757). The mixture was placed in a thermocycler (PTC 200, MJResearch, Biozym Diagnostic, Hess, Oldendorf, Germany), heated for 1 min at 94°C, and subjected to 30 cycles of 20 s denaturation at 94°C, 20 s each for the first annealing reaction at 63°C with a 40 s extension at 72°C and a final extension for 2 min at 72°C. After the first amplification with the outer set of primers, 2 μL of the amplification product was transferred to a fresh tube containing 48 μL of the reaction mixture previously described, except that 2.5 mM MgCl_2_ and 20 pmol of the inner primer pair was used (16S2A 5′-AGT CAA ACG GGA TGT AGC AAT AC-3′ and 16S2B 5′-GGT ATT CTT TCT GAT ATC AAC AG-3′ positions 66–720). This mixture was subjected to 35 amplification cycles by using the cycle conditions described previously, except that the annealing reaction was performed at 56°C and the extension reaction lasted 30 s. DNA was extracted, reaction vials were prepared for amplification, templates were added, and products underwent electrophoresis in separate rooms. In each sixth reaction mix, water was added instead of extracted DNA to serve as negative control. PCR products were detected by electrophoresis in a 1.5% agarose gel stained with ethidium bromide.

Each PCR amplification product was purified by using a QIAquick-Spin PCR column (Qiagen QmbH) according to the manufacturer’s instructions. Amplified DNA fragments were directly sequenced in both directions by using the inner primers by the dideoxynucleotide chain-termination method on a Li-COR DNA4200 sequencer (Li-COR Biosciences, Bad Homburg, Germany). Each resulting sequence was compared with sequences of the same gene fragment representing various spirochetal genospecies. The following sequences served for comparison: accession nos. X85196 and X85203 for *B. burgdorferi* s.s.; X85190, X85192, and X85194 for *B. afzelii*; X85193, X85199, and M64311 for *B. garinii*; X98228 and X98229 for *B. lusitaniae*; AJ224138 for *B. bissettii*; X98232 and X98233 for *B. valaisiana*; AY024345 for the American *Borrelia* sp.nov.; and D45192 for *B. miyamotoi*. A complete match, permitting no more than two nucleotide changes, was required.

For comparison with the American and Asian relapsing fever–like spirochetes, *Borrelia* genus–specific primers (Bf1 5′-GCT GGC AGT GCG TCT TAA GC-3′ and Br1 5′-GCT TCG GGT ACT CTC AAC TC-3′) were used to amplify and sequence a fragment of the 16S rRNA gene approximately 1,350-bp in length, according to the published PCR conditions (*15*). A set of degenerate primers (FLA120F 5′-AGA ATT AAT MGH GCW TCT GAT GAT G-3′ and FLA920R 5′-TGC YAC AAY HTC ATC TGT CAT T-3′) was used to amplify and sequence an approximately 800-bp fragment of the flagellin gene, according to the published PCR conditions (*12*).

## Results

To determine whether relapsing fever–like spirochetes may infect *I. ricinus* ticks in our study sites, DNA of field-derived nymphs and adults was amplified by using primers that bind to a 16S rRNA gene fragment present in relapsing fever–like spirochetes as well as Lyme disease spirochetes. Of the 565 ticks that were processed, 197 produced amplification products. Of these, the sequences of 177 were consistent with those of known genospecies of the Lyme disease spirochete. Derived sequences from the remaining 20 samples corresponded closely to sequences of the Asian and American relapsing fever–like spirochetes. These DNA fragments (GenBank accession no. AY253149) differed by six bases from the American and Asian relapsing fever–like spirochetes. In all three sites, DNA of bacteria related to relapsing fever spirochetes was detected in questing *I. ricinus* ticks.

We estimated the prevalence of the various spirochetal infections in ticks sampled in our study sites. Spirochetes infected approximately a quarter of nymphal and more than a third of adult ticks (Table 1); adults were infected more frequently than were nymphs (p=0.0001, Fisher exact test). Approximately 3.5% of these questing *I. ricinus* ticks were infected by a relapsing fever–like spirochete, constituting approximately one tenth of all spirochete-infected ticks (10.2%). The prevalence of infection by the relapsing fever–like spirochetes was similar at each of the three sites. Although the relapsing fever–like spirochetes appeared to infect more adult than nymph ticks, this difference was not significant. A relapsing fever–like spirochete commonly infects questing *I. ricinus* ticks in central Europe.

**Table 1:** Prevalence of spirochetal variants in questing *Ixodes ricinus* ticks sampled from central Europe^a^

Site	Ticks examined			% infected ticks infected by *Borrelia*						% total ticks infected by RFS
	stage	no.	% infected	RFS	afz	gar	val	bur	lus	
PC	nymph	107	26.2	10.7	28.6	28.6	32.1	7.1	0	2.8
	adult	34	52.9	16.7	33.3	22.2	27.8	5.6	0	8.8
MK	nymph	63	28.6	5.6	27.8	55.6	5.6	11.1	0	1.6
	adult	113	38.9	13.6	22.7	31.8	29.5	2.3	0	5.3
LB	nymph	45	17.7	0	62.5	12.5	0	0	25.0	0
	adult	203	39.9	8.6	37.0	25.9	7.4	1.2	22.2	3.4

^a^The sampling sites were Petite Camargue Alsacienne, France (PC), Maikammer, Germany (MK), and Lembach, France (LB), and the variants were *B. afzelii* (afz), *B. garinii* (gar), *B. valaisiana* (val), *B. burgdorferi* s.s. (bur), and *B. lusitaniae* (lus), European relapsing fever–like spirochete (RFS).

The relatedness of the European relapsing fever–like spirochete to such spirochetes from Asia and North America was determined by comparing a 780-bp fragment of the flagellin gene as well as a 1,106-bp fragment of the 16S rRNA gene. The flagellin sequence of the European relapsing fever–like spirochete (GenBank accession no. AF529084) was 98.8% similar to that of the American spirochete and 98.3% similar to that from Japan (Table 2). The European spirochetes were characterized by six unique nucleotides. The 16S rDNA sequence of the European variant (GenBank accession no. AF529085), similarly, shared 99.6% and 99.5% of the sequence of the North American and Japanese spirochete, respectively (Table 3). The flagellin and 16S rDNA sequences of the various European relapsing fever–like spirochetes were identical. The European relapsing fever–like spirochete differs from its American and Asian relatives and is relatively homogeneous within the region.

**Table 2:** Similarity matrix for the flagellin sequences of various relapsing fever and relapsing fever–like spirochetes (RFS)

Species	Variant	Accession no.	% similarity with					
			American RFS	European RFS	*Borrelia miyamotoi*	*B. lonestari*	*B. hispanica*	“Spain” strain
American RFS	MP2000	AY024344	100					
European RFS	LB-M56	AF529084	98.84	100				
*B. miyamotoi*	HT31	D43777	98.56	98.32	100			
*B. lonestari*	Texas	U26704	89.66	89.17	89.98	100		
*B. hispanica*	N.n.^a^	U28498	85.60	84.90	85.82	84.36	100	
“Spain” strain	N.n.	U28499	88.35	88.06	88.94	86.86	94.32	100

^a^N.n., not named.

**Table 3:** Similarity matrix for the 16S rRNA sequences of various relapsing fever and relapsing fever–like spirochetes (RFS)

Species	Variant	Accession no.	% similarity with					
			American RFS	European RFS	*Borrelia miyamotoi*	*B. lonestari*	*B. hispanica*	“Spain” strain
American RFS	MP2000	AY024345	100					
European RFS	LB-W100	AF529085	99.64	100				
*B. miyamotoi*	HT31	D45192	99.34	99.46	100			
*B. lonestari*	Texas-20	U23211	98.19	98.28	98.19	100		
*B. hispanica*	UESV/246	U42294	98.16	98.37	97.79	98.13	100	
“Spain” strain	N.n.^a^	U28502	97.10	97.56	96.69	97.06	98.87	100

^a^N.n., not named.

We described the relative prevalence of each genospecies in our sample of ticks. *B. afzelii* and *B. garinii* were most prevalent, each infecting approximately a third of all spirochete-infected ticks, regardless of location (Table 1). *B. burgdorferi* s.s., in contrast, infected few such ticks. The prevalence of *B. valaisiana* varied among our study sites; this spirochete appeared to be more prevalent in the sites that were considered southern (p<0.05, Fisher exact test). The range of *B. lusitaniae* was more restricted than that of the other genospecies. The genospecies of Lyme disease spirochetes that are most commonly detected in central Europe are distributed broadly, whereas those that are found less frequently tend to be place-specific.

Finally, we determined how frequently ticks might be infected by both relapsing fever–like and Lyme disease spirochetes. Of the 565 questing ticks examined, 7 (1.2%) harbored more than one spirochete variant. Mixed infections were distributed similarly among our study sites. *B. garinii* and *B. valaisiana* appeared to be associated with each other, co-infecting three adult ticks, as were *B. afzelii* and *B. burgdorferi* s.s., co-infecting two nymphs and one adult. Another adult tick harbored *B. garinii* and *B. afzelii*. No co-infections of relapsing fever–like and Lyme disease spirochetes were noted. The absence of an association between relapsing fever–like spirochetes and Lyme disease spirochetes in ticks suggests that these spirochetes may not share common hosts.

## Discussion

Among the many microbes transmitted by the vectors of Lyme disease, spirochetes are distinct *Borrelia* related to relapsing–fever spirochetes. *B. miyamotoi* infects *I. persulcatus* ticks in Japan, and a closely related spirochete infects a related tick on the East Coast of North America (*10*,*12*). We now know that a third member of this group infects *I. ricinus* ticks in central Europe. Despite their wide distribution, the three relapsing fever–like spirochetes appear to vary little; few nucleotides are substituted in the sequences of the two gene fragments that were examined. The relapsing fever–like spirochetes appear to be homogeneous in North America (*12*) as well as in Europe. We conclude that each of the various kinds of ticks that serve as vectors for Lyme disease spirochetes, *I. ricinus*, *I. persulcatus*, *I. scapularis* (= *dammini*), may be infected by relapsing fever–like spirochetes.

The recently identified relapsing fever–like spirochetes appear to co-exist regionally with the agents of Lyme disease and may inflate estimates of exposure risk to Lyme disease spirochetes on the basis of their prevalence in vector ticks. Neither darkfield nor immunofluorescence microscopy serves to discriminate Lyme disease spirochetes from relapsing fever–like spirochetes (*12*). By amplifying a particular fragment of the 16S rRNA gene, Lyme disease spirochetes may be distinguished from relapsing fever–like spirochetes and identified to the genospecies level. Relapsing fever–like spirochetes constitute approximately one tenth of spirochetes infecting North American vector ticks (*12*) as well as those in central Europe. Questing *I. ricinus* ticks in our study sites more frequently harbor relapsing fever–like spirochetes than *B. burgdorferi* s.s. (p<0.05, Fisher exact test). The proportion of relapsing fever–like spirochetes appears to be similar in the population of European and American vector ticks.

The vertebrate host associations of relapsing fever–like spirochetes transmitted by hard ticks have not yet been defined. If the prevalence of a tick-borne pathogen increases with each developmental stage of the tick, a reservoir host is likely involved in its transmission cycle. Although evidence of such a pattern of accumulation, in the case of the relapsing fever–like spirochetes, is no more than marginal; field, as well as laboratory, observations suggest the possibility of vertebrate hosts. *B. miyamotoi* has been isolated from the blood of a Small Japanese Field Mouse (*Apodemus argenteus*) (*10*). Infectivity of such naturally infected animals to ticks, however, has not been demonstrated. White-footed Mice (*Peromyscus leucopus*) experimentally infected by tick bites have occasionally infect other ticks (*12*). Relapsing fever–like spirochetes are transmitted less frequently than Lyme disease spirochetes that cycle between the same reservoir and vector. Perpetuation of the relapsing fever–like spirochetes may not require a vertebrate reservoir.

To the extent that diverse pathogens share reservoir hosts, they might also co-infect their vector hosts. Although various Lyme disease spirochetes occasionally are detected in the same tick, the relapsing fever–like spirochetes appear not to co-infect ticks that are infected by Lyme disease spirochetes. None of our 565 European ticks and only 1 of 712 American ticks in an earlier study were co-infected in this manner (*12*). If these infections were distributed randomly, numerous co-infections would be anticipated. The presence of one kind of spirochete, however, appears to exclude the presence of the other. Lyme disease and relapsing fever–like spirochetes may directly interact in their tick or vertebrate hosts (e.g., inhibiting the other’s proliferation) or each may be closely associated with a reservoir host that is zooprophylactic for the other kind of spirochete. Co-infection between diverse spirochetes occurs only infrequently.

The distribution of *B. lusitaniae*, the most recently described Lyme disease genospecies, is anomalous. With the possible exception of *B. valaisiana*, the prevalence of the other genospecies is constant from site to site. Indeed, these estimates correspond closely to those in a survey conducted in more northerly sites (*16*,*17*). *B. lusitaniae*, in contrast, was detected in only one of the three sites surveyed in the present study and not at all in four other sites that we have investigated (unpub. data). Although this genospecies may more frequently infect ticks in the Mediterranean basin (Portugal [*18*], Spain [*19*], and Tunisia [*20*]), it has been detected in ticks as far east as Slovakia (*21*), Moldovia, the Czech Republic, and Ukraine (*22*). The prevalence of *B. lusitaniae* in our Lembach site corresponds more to that in southern than in eastern Europe. Although *B. lusitaniae* induces disease when injected into susceptible mice (*23*), nothing is known about its pathogenic potential in people. The distribution of *B. lusitaniae* is irregular and its biologic relationships largely unknown.

Certain members of the relapsing fever group of spirochetes, mainly those transmitted by soft ticks, cause severe human disease. The classic agent of human relapsing fever in Europe is attributed to infection by *B. hispanica,* a relatively rare *Ornithodoros-*transmitted spirochete endemic to the southern part of that continent (*4*). Recently, a “Spain strain” of such spirochetes was implicated in human disease (*24*). The relapsing fever–like spirochetes that we describe differ fundamentally from these pathogens in that they are transmitted by hard ticks. Two kinds of spirochetes in this group are endemic to North America, *B. theileri* and *B. lonestari*, which are transmitted mainly by *Boophilus* and *Amblyomma* ticks, respectively. *B. theileri* is the agent of bovine borreliosis, a severe veterinary disease (*25*). *B. lonestari* appears to cause a human illness, known as Master’s disease or southern tick-associated rash illness (STARI) (*26*–*29*). Although the Asian, American, and European *Ixodes*-borne relapsing fever–like spirochetes relate more closely to each other than to *B. theileri* and *B. lonestari*, they form a monophyletic clade with these other hard-tick–associated spirochetes (*12*,*30*). Neither the pathognomonic recurrent fever of the soft-tick–transmitted spirochetes nor the transient fever and rash associated with Master’s disease has been associated with infection by the *Ixodes-*borne relapsing fever–like spirochete. Exposure risk of this agent is almost as great as that to one or another of the European Lyme disease genospecies. Although many people may be bitten by ticks infected by relapsing fever–like spirochetes, the resulting health implications remain unknown.
